# Subpopulations of hyphae secrete proteins or resist heat stress in *Aspergillus oryzae* colonies

**DOI:** 10.1111/1462-2920.14863

**Published:** 2019-11-24

**Authors:** Martin Tegelaar, Robert‐Jan Bleichrodt, Benjamin Nitsche, Arthur F. J. Ram, Han A. B. Wösten

**Affiliations:** ^1^ Microbiology Utrecht University Padualaan 8 3584 CH Utrecht The Netherlands; ^2^ Molecular Microbiology and Biotechnology Leiden University Sylviusweg 72 2333 BE Leiden The Netherlands

## Abstract

Hyphae at the outer part of colonies of *Aspergillus niger* and *Aspergillus oryzae* are heterogeneous with respect to transcriptional and translational activity. This heterogeneity is maintained by Woronin body mediated closure of septal pores that block interhyphal mixing of cytoplasm. Indeed, heterogeneity between hyphae is abolished in Δ*hexA* strains that lack Woronin bodies. The subpopulation of hyphae with high transcriptional and translational activity secretes enzymes that degrade the substrate resulting in breakdown products that serve as nutrients. The role of hyphae with low transcriptional and translational activity was not yet known. Here, we show that this subpopulation is more resistant to environmental stress in *A*. *oryzae*, in particular to temperature stress, when compared to hyphae with high transcriptional and translational activity. Notably, all hyphae of the Δ*hexA* strain of *A*. *oryzae* were sensitive to heat stress explained by the reduced heterogeneity in this strain. Together, we show that different subpopulations of hypha secrete proteins and resist heat stress showing the complexity of a fungal mycelium.

## Introduction

Hyphae of filamentous fungi grow at their tips and branch subapically, which gives rise to a network of hyphae called mycelium. Mycelia are heterogeneous with respect to gene expression, growth and secretion (Wösten *et al*., 2013). For instance, only part of the hyphae at the periphery of the mycelium of *Aspergillus niger* secrete glucoamylase (Wösten *et al*., 1991) and express its encoding gene (Vinck *et al*., 2005; de Bekker *et al*., 2011). Hyphae with high *glaA* expression also highly express other genes encoding secreted proteins. Moreover, they have a high rRNA content and highly express the glyceraldehyde‐3‐phosphate dehydrogenase gene *gpdA* (Vinck *et al*., 2011). From these studies, it was concluded that a subpopulation of hyphae with ‘high’ and a subpopulation of hyphae with ‘low’ transcriptional and translational activity can be distinguished at the outer part of the vegetative mycelium. Low activity would be sufficient to support growth but high activity would be needed to support secretion of high amounts of protein.

Hyphae of fungi belonging to the Ascomycota and the Basidiomycota are compartmentalized by porous septa. The diameter of the septal pore varies between 50 and 500 nm allowing passage of cytosol and even organelles (Shatkin and Tatum, 1959; Moore and McAlear, 1962; Gull, 1978; Lew, 2005). The septal pores of ascomycetes can be closed by peroxisome‐like organelles called Woronin bodies. Deletion of *hexA* in *Neurospora crassa*, *Magnaporthe grisea*, *Aspergillus oryzae* and *A*. *niger* results in the absence of Woronin bodies. This abolishes closure of septa after hyphal damage (Jedd and Chua, 2000; Tenney *et al*., 2000; Soundararajan *et al*., 2004; Maruyama *et al*., 2005) and results in excessive cytoplasmic bleeding. Woronin bodies can also plug septa of intact growing hyphae (Markham, 1994; Bleichrodt *et al*., 2012, 2015a; Steinberg *et al*., 2017a,b). Plugging of these pores has been shown to block intra‐ and intercompartmental cytoplasmic mixing (Bleichrodt *et al*., 2012, 2015a). Consequently, heterogeneity in composition of hyphae and their compartments and hyphae is maintained (Bleichrodt *et al*., 2012, 2015a).

So far, it has been shown that the subpopulation of hyphae that has high transcriptional and translational activity highly secrete proteins. A role of the lowly active hyphae was not yet clear. In this study, it is shown that these hyphae of *A*. *oryzae* are more resistant to environmental stress, in particular to heat. Together, it is concluded that subpopulations of hyphae at the outer part of *A*. *oryzae* colonies have different functions.

## Results

### *Environmental conditions affect septal plugging in* A. niger *and* A. oryzae

**Figure 1:**
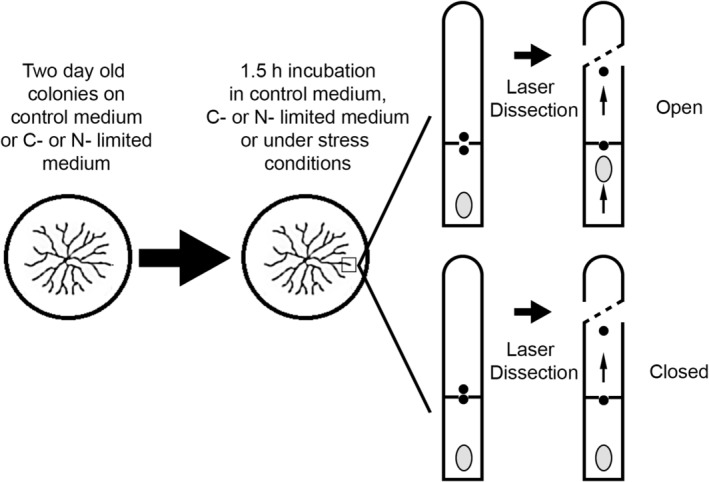
Schematic representation of the way stress was induced in fungal colonies and how septal closure was scored. Septa were scored as being open when movement of organelles (grey ellipses) towards the septum was observed after laser dissection of the subapical compartment. Black dots represent Woronin bodies and their presumed location. Laser dissection in this example is performed in the apical compartment. The location of the disruption is represented by a dashed line.

**Figure 2:**
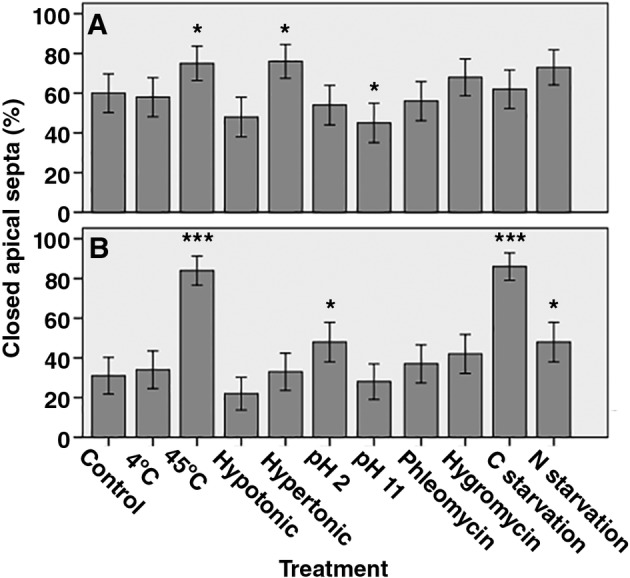
Percentage of closed apical septa of growing hyphae at the periphery of *A*. *niger* N402 (A) and *A*. *oryzae* RIB40 (B) colonies that had been subjected to different stress conditions. Error bars indicate 95% CIs. Asterisks (* p ≤ 0.05, ** p ≤ 0.01, *** p ≤ 0.001) indicate significant differences between control conditions and treatment conditions. N = 100.

Hyphae at the periphery of fungal colonies explore the non‐colonized substrate. This substrate can be heterogeneous with respect to physical (e.g., temperature, osmotic potential) and chemical (e.g., presence or absence of C‐ or N‐source or antibiotics secreted by other microbes in the substrate) conditions. The effect of environmental conditions on plugging of the apical septum of leading hyphae of *A*. *niger* and *A*. *oryzae* was assessed. To this end, wild‐type strains N402 and RIB40 were grown for 2 days at 30 °C, after which they were either or not exposed to temperature stress, pH stress, hypo‐ or hypertonic conditions, 25 μg ml^−1^ phleomycin or 4 mg ml^−1^ hygromycin. Alternatively, strains were starved for 2 days for carbon or nitrogen. Septal plugging was assessed by laser dissection of the apical compartment (Fig. 1 and *Experimental procedures*). Significant changes in plugging incidence of the most apical septa were found when *A*. *oryzae* and *A*. *niger* were exposed to 45 °C for 1.5 h (Fig. 2). This temperature stress increased plugging incidence from 60% to 75% and 31% to 83% in the case of *A*. *niger* and *A*. *oryzae* respectively. Additionally, plugging incidence of apical septa in *A*. *niger* increased to 75% under hypertonic conditions and decreased to 45% when exposed to high pH. In the case of *A*. *oryzae*, apical plugging incidence increased to 48%, 86% and 48% under low pH conditions, C‐starvation and N‐starvation respectively. Together, these data show that environmental conditions affect septal closure of *A*. *niger* and *A*. *oryzae*. Yet, their response to such conditions is different.

### *Growth of* A. niger *and* A. *oryzae* ΔhexA *strains can be affected by environmental conditions*

The diameter of 24 h old colonies of control and Δ*hexA* strains of *A*. *niger* (N402 and N402ΔhexA, respectively) and *A*. *oryzae* (RB#153.1 and RB#149.1, respectively) was measured after growing at standard conditions or after carbon starvation. Colony diameter was 2 mm for all strains at standard growth conditions. This was also the case for the *A*. *niger* and *A*. *oryzae* control strains that had been grown under carbon limitation. In contrast, colony diameter of *A*. *niger* and *A*. *oryzae* Δ*hexA* strains was 2.4 mm and 0.6 mm, respectively, after 24 h of carbon starvation. This shows that growth of the *A*. *oryzae* Δ*hexA* strain but not the wild‐type strain is affected by carbon starvation.

**Figure 3:**
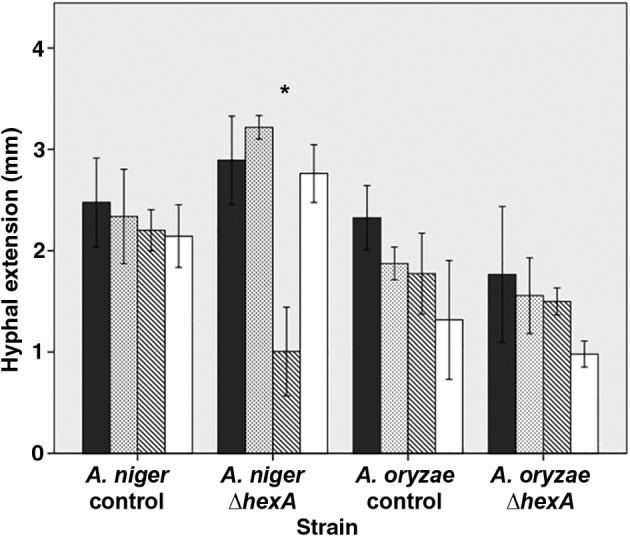
Hyphal extension (mm) of 24 h old control (N402, RB#153.1) and Δ*hexA* (N402Δ*AnhexA*, RB#149.1) strains that had either or not (black bars) been exposed to hypertonic conditions (dotted bars) or 45 °C (diagonal stiped bars) for 1,5 h and subsequently allowed to recover for 24 h under non‐stress conditions. White bars denote colonies that had been exposed to C‐starvation for 25.5 h, after which growth was prolonged for 24 h under standard growth conditions. Error bars indicate standard deviations. Asterisk (*p* ≤ 0.05) indicates significant differences from control conditions within strains.

In the next step, 24 h old *A*. *niger* and *A*. *oryzae* colonies were either or not exposed for 1.5 h to stress conditions, which was followed by incubation for 24 h under normal growth conditions. Hyphae of the wild‐type *A*. *niger* colonies had extended by 2.3 mm during the 25.5 h period, irrespective whether they had been exposed to stress (Fig. 3). The *A*. *niger* Δ*hexA* strain showed similar growth except for hyphae that had been exposed to 45 °C for 1.5 h. These hyphae had only extended by 1 mm. No significant differences were observed in the case of *A*. *oryzae*, irrespective of strain or heat treatment (Fig. 3). Together, this shows that *A*. *oryzae* can restore its growth rate after exposure to stress, if affected at all, irrespective of the presence of *hexA*. In contrast, sensitivity of *A*. *niger* Δ*hexA* cannot be restored by growing at standard growth conditions.

### *Hyphal heterogeneity promotes adaptability of the colony to stress conditions*

**Figure 4:**
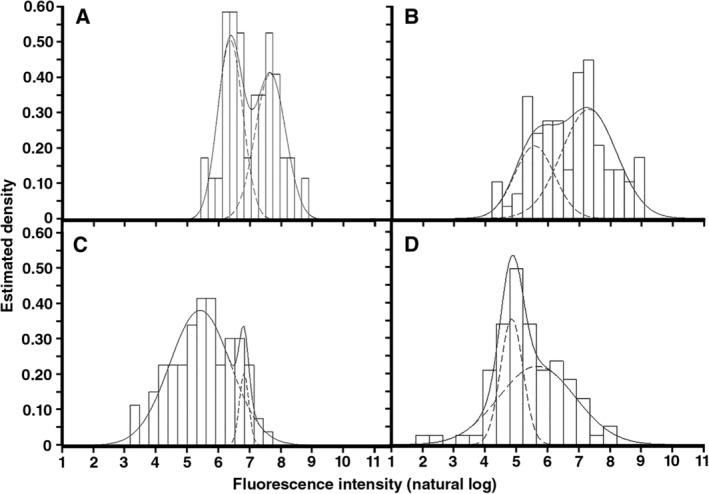
Estimated density plots based on normalized natural logarithm transformed fluorescence intensity data of hyphae of RB#140.1 (A, B) and RB#141.3 (C, D) that had either (B, D) or not (A, C) been exposed to 45 °C for 90 min. The former strain has Woronin bodies, while the latter does not.

*Aspergillus oryzae* was shown to recover from heat stress by growing for 24 h at standard growth conditions (see above). As a next step, growth of hyphae and *glaA*‐driven green fluorescent protein (GFP) fluorescence was measured immediately after heat shock. This was done with the control (RB#140.1) and the Δ*hexA* (RB#141.3) strains that express *gfp* from the glucoamylase *glaA* promoter. Expression of *glaA* was induced by transfer to maltose containing medium. This was followed by either or not exposing the colonies at 45 °C for 1.5 h. Expression of *glaA* within hyphae at the periphery of colonies of the *A*. *oryzae* control and Δ*hexA* strain were best described by a bimodal distribution when grown under control conditions. The fraction of high fluorescent hyphae was 50% and 7.5% for the control and Δ*hexA* strain respectively. After heat shock, heterogeneity was reduced in the *A*. *oryzae* control strain and abolished in the Δ*hexA* strain (Fig. 4).

**Figure 5:**
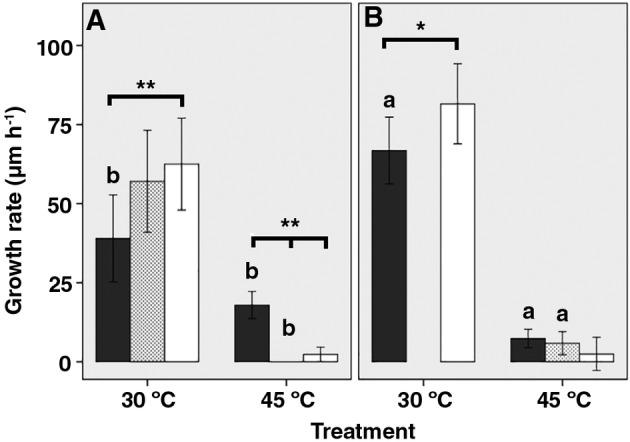
Highly (black bars), intermediate (white dotted bars) and lowly (white bars) *glaA* expressing hyphae of the *A*. *oryzae* control strain RB#140.1 (A) and the Δ*hexA* strain RB#141.3 (B) grown at 30 °C or 45 °C. Statistics did not indicate an intermediate population of fluorescent hyphae in the case of the Δ*hexA* strain grown at 30 °C. Error bars represent 95% CIs. Lower case a and b indicate significant differences in growth rate for fluorescence intensity levels between strains. Asterisks (*p ≤ 0.05, **p ≤ 0.01) and lines indicate significant differences in growth rate for hyphae with different fluorescence intensity levels within treatments. All growth rates of hyphae with different fluorescence intensity levels are significantly different between treatments.

Mean fluorescence intensity of hyphae at the colony periphery had reduced 90 min after heat‐shock from 958 to 844 and 262 to 234 arbitrary units for the *A*. *oryzae* control and Δ*hexA* strain respectively. No reduction in fluorescence intensity was observed for colonies exposed to control conditions. Low fluorescent hyphae of the control and the Δ*hexA* strain that had not been exposed to 45 °C had grown 39 and 67 μm h^−1^, respectively, while the high GFP expressing hyphae had extended 63 and 82 μm h^−1^ (Fig. 5). Growth of heat stress exposed hyphae that showed low GFP fluorescence was reduced to 18 and 7 μm h^−1^ in the case of the control strain and the Δ*hexA* strain respectively. In contrast, extension of hyphae that were highly fluorescent was reduced to 2 μm h^−1^ for both strains. Together, hyphae of the control *A*. *oryzae* strain that highly express *glaA* are more sensitive to heat stress than hyphae lowly expressing *glaA*. Furthermore, both lowly and highly expressing hyphae of the Δ*hexA* strain showed faster growth under control conditions, while they were more sensitive to temperature stress. This implies a trade‐off between fast growth and adaptability to environmental stress.

## Discussion

A subpopulation of hyphae with low transcriptional and translational activity and a subpopulation of hyphae with high such activities are found at the outer part of colonies of *A*. *oryzae* and *A*. *niger* (Vinck *et al*., 2005, 2011; Bleichrodt *et al*., 2015a,b). The highly active hyphae secrete proteins, while we here show that the lowly active hyphae are more resistant to environmental stress, in particular to high temperature stress.

Woronin bodies maintain the heterogeneity of hyphae at the outer part of the *Aspergillus* colony (Bleichrodt *et al*., 2012). As a consequence, GFP fluorescence intensity distribution resulting from *glaA* driven expression is heterogeneous (i.e., bimodal) between hyphae at the periphery of wild‐type colonies but homogenous (i.e., unimodal) between Δ*hexA* hyphae that lack Woronin bodies. Here, we did find a bimodal GFP fluorescence intensity distribution for the Δ*hexA* strain. Yet, the subpopulation of the highly active hyphae was very small (only 7.5% of the total hyphae), while this subpopulation represents half the hyphae in the wild type. Thus, our data also support the role of Woronin bodies in maintenance of hyphal heterogeneity.

Part of the septa of vegetative hyphae of the basidiomycetes *Schizophyllum commune* and *Rhizoctonia solani* and the ascomycetes *A*. *oryzae*, *A*. *niger* and *Zymoseptoria tritici* are closed (van Driel *et al*., 2008; van Peer *et al*., 2009, 2010; Bleichrodt *et al*., 2012, 2015a; Steinberg *et al*., 2017b). Incidence of septal closure in vegetative growing hyphae of *S*. *commune* depends on environmental conditions. Heat shock, hypertonic shock and presence of antibiotics promote septal plugging, while low glucose reduces plugging incidence (van Peer *et al*., 2009). Such an effect was not observed in *A*. *oryzae* (Bleichrodt *et al*., 2012). Here, we did show an effect of environmental conditions on septal plugging both for *A*. *niger* and *A*. *oryzae*. This is due to the use of a larger sample size and adapted statistical analysis. Plugging incidence of the apical septa of leading hyphae of *A*. *oryzae* was affected by heat treatment, low pH conditions, C‐starvation and N‐starvation. On the other hand, plugging incidence in *A*. *niger* was affected by exposure to heat, high pH and hypertonic conditions. Together, these data indicate that closure of septa in *Aspergillus* is not a stochastic process but a result of environmental conditions. Results also show that *A*. *oryzae* and *A*. *niger* respond differently to these conditions. This may be caused by the limits of the environmental conditions that allow growth of these aspergilli (Krijgsheld *et al*., 2013). For instance, *A*. *niger* can still grow at pH 1.5, while growth of *A*. *oryzae* only occurs above pH 4.

*Aspergillus niger* and *A*. *oryzae* strains with Woronin bodies were not affected in growth during carbon starvation. In contrast, growth of the *A*. *oryzae* Δ*hexA* strain was severely affected. Hyphal extension of the *A*. *niger* and *A*. *oryzae* Δ*hexA* strains was also affected by heat treatment. This effect was only found directly after the treatment in the case of *A*. *oryzae* Δ*hexA*, while in the case of the *A*. *niger* Δ*hexA* strain the effect of temperature was even observed after 24 h of recovery. It may well be that septal closure mediated hyphal heterogeneity is also instrumental in other environmental stresses. Deletion of a gene encoding the Woronin body associated transmembrane protein TmpL in *Alternaria brassicicola* and *A*. *fumigatus* results in hypersensitivity to oxidative stress (Kim *et al*., 2009). Moreover, a Δ*HEXA* strain of *M*. *grisea* is not able to survive N‐starvation (Soundararajan *et al*., 2004). In addition, *hexA* is upregulated in *Trichoderma atroviride* stressed by the organophosphate pesticide dichlorvos (Tang *et al*., 2010).

Lack of Woronin bodies not only abolishes hypal heterogeneity, but also increases growth rate. Tegelaar and Wösten (2017) found that wild‐type *A*. *niger* hyphae grow at 84% of the growth rate of the *A*. *niger* Δ*hexA* strain. Here, a similar effect was shown for *A*. *oryzae*. Hyphae with high transcriptional and translational activity grew 23% slower than the Δ*hexA* strain, while this was even 42% in the case of lowly active hyphae. Our data thus suggest that fast growth comes with a reduced ability to respond to environmental stress. Together, it is concluded that the outer part of a wild‐type *A*. *oryzae* colony consists of hyphae that are specialized in release of nutrients from the substrate by secreting proteins and hyphae that have higher stress resistance. Why would *Aspergillus* use this strategy? Organisms that live in a heterogeneous environment must adapt to survive. A clonal population of cells such as that in a mycelium can employ two main strategies to adapt. First, (phenotypically uniform) individuals can sense their environment followed by immediate switching their phenotype to the one most suited to the new condition. Alternatively, phenotypically diverse individuals colonize the substrate, of which some are not optimally suited for their current environment but may be well adapted to a potential change in environment, a phenomenon known as bet‐hedging (Kussell and Leibler, 2005; Zacchetti *et al*., 2018). When the environment changes frequently, the former strategy is preferred, while the latter strategy is preferred when cells are exposed to infrequent changes. In nature, *Aspergillus* hyphae are exposed infrequently to environmental changes in relation to the length of their life cycle, being a few days. Indeed, we here showed a bet‐hedging strategy for *Aspergillus* hyphae with respect to heat. Together, it is concluded that the existence of a subpopulation of persister‐type hyphae at the periphery of the *Aspergillus* colony ensures efficient colonization of substrates.

## Experimental procedures

### *Strains, media and spore isolation*

Strains used in this study (Table 1) were grown on minimal medium (MM) (de Vries *et al*., 2004) with 200 mM xylose or 25 mM maltose as a carbon source or on CD + Met medium (Maruyama *et al*., 2010) supplemented with 1% glucose. Spores were harvested from 3 day old cultures that had been grown on complete medium (MM with 0.2% trypton, 0.1% casamino acids, 0.1% yeast extract, 0.05% yeast ribonucleic acids and 1% glucose) using saline tween [ST, 0.9% NaCl (wt/vol), 0.05% (vol/vol) Tween‐20].

**Table 1:** Strains used in this study.

Strain	Parental strain	Genotype	Reference
*A*. *oryzae* RIB40		Wild‐type	Machida *et al*. (2005)
*A*. *niger* N402	NRRL 3	Δ*cspA1*	Bos *et al*. (1988)
*A*. *niger* N402Δ*hexA*	N402	Δ*cspA1* Δ*hexA hygR+*	This study
*A*. *niger* RB65.2	N402Δ*hexA*	Δ*cspA1* Δ*hexA hygR+ AnhexA phleoR+*	This study
*A*. *oryzae* RB#140.1	NSRKu70‐1‐1AS	Δ*ku70 glaA::sGFP niaD+*	Bleichrodt *et al*., 2012
*A*. *oryzae* RB#141.3	NSRK‐ΔHx5	Δ*ku70* Δ*Aohex1 glaA::sGFP niaD+*	Bleichrodt *et al*. (2012)
*A*. *oryzae* RB#153.1	NSRKu70‐1‐1AS	Δ*ku70 niaD* ^*+*^	This study
*A*. *oryzae* RB#149.1	NSRK‐ΔHx5	Δ*ku70* Δ*Aohex1 niaD* ^*+*^	This study

### *Construction of* A. oryzae *strains*

NSRKu70‐1‐1AS and NSRK‐ΔHx5 were transformed with pNR10 harbouring the *niaD* gene (Yoon *et al*., 2010). The resulting strains RB#153.1 and RB#149.1 are prototrophic for nitrate.

### *Construction of* A. niger *strains*

To inactivate *hexA* (An07g04570) of *A*. *niger*, its 0.9 kb upstream and 1.0 kb downstream flanking regions were amplified with primer pairs BN090 and BN091 and BN088 and BN089 (Supplementary Table 1), respectively, using genomic DNA of strain N402 as a template. The up‐ and down‐stream sequences were inserted by BP recombination into pDONR™ P4‐P1R and pDONR™ P2R‐P3 (Invitrogen, http://www.invitrogen.com), respectively, generating the 5′ and 3′ entry clone plasmids pDONR_P‐HexA and pDONR_T‐HexA. The hygromycin resistance cassette (*hygR*) was amplified with primers hygroFW and hygroREV (Table S1) using pAN7.1 (Punt *et al*., 1987) as a template and inserted into pDONR™ P1‐P2R (Invitrogen) by BP recombination generating the centre entry clone plasmid pDONR_hygR. The 5′, 3′ and centre entry constructs were subjected to LR clonase in the presence of pDEST R4‐R3 (destination vector) to obtain the final plasmid pΔ*AnHexA*. The DNA fragment including the *hexA* flanking regions interspersed by the hygromycin resistance cassette was amplified by poly chain reaction (PCR) using primers BN090 and BN089 and plasmid pΔ*AnHexA* as a template and was introduced into *A*. *niger* strain N402, generating strain N402Δ*hexA*. Disruption of the *hexA* gene was confirmed by Southern blotting using genomic DNA that had been digested with BamHI and KpnI.

To complement the N402Δ*hexA* strain, the *hexA* gene was amplified by PCR with primers AnhexA‐FW‐NotI (TAGCTATAGCGGCCGCAGTTGATCTAGCGCGTGAACG) and AnhexA‐REV‐PstI (TCGCTATACTGCAGTTACGACGGCACGAAACGGC). The PCR product was digested with restriction enzymes NotI and PstI and introduced in plasmid pRB206 (Wang *et al*., 2015) that had been cut with the same enzymes. This resulted in plasmid pB065 that contains the *hexA* gene and a phleomycin resistance cassette. pB065 was transformed to the Δ*hexA* strain. Complementation of *hexA* was assessed by chromosomal DNA extraction and PCR with primers ORF‐hexA‐FW (ATGGGTTACTACGACGACGACG) and ORF‐hexA‐REV (CATCCTCGAAGGCCTCACGG). Chromosomal DNA from N402 and *ΔhexA* strains was used as a control. PCR was positive for transformants RB65.2, RB65.3, RB65.4 and negative for the *ΔhexA* strain. Sequencing of the PCR bands confirmed *hexA* identity. To confirm phenotypic complementation, septal plugging of apical septa was assessed by laser microdissection. When the complemented strain RB#65.2 was grown under control conditions, 12% of apical septa were closed when cut in the apical compartment, thus confirming the strain was complemented.

### *Transformation of* Aspergillus

Protoplasts of *A*. *niger* and *A*. *oryzae* were generated as described (de Bekker *et al*., 2009) and transformed using polyethylene glycol (Punt and van den Hondel, 1992). Nitrate prototrophic (*niaD* ^+^) strains of *A*. *oryzae* were selected on MMS medium (minimal medium pH 6.0, 0.95 M sucrose and 1.5% agar). Transformants of *A*. *niger* were selected on MMS medium containing 100 μg ml^−1^ hygromycin or 25 μg ml^−1^ phleomycin (InvivoGen; http://www.invivogen.com).

### *Quantification of septal plugging*

*Aspergillus* strains were grown under water saturating conditions in 35 mm glass bottom dishes (MatTek Corporation, Ashland, MA, P35G‐1.5‐20‐C) (Bleichrodt *et al*., 2012). To this end, 30 μl 60 °C CD + Met medium with 1% glucose and 1% agarose (CDMGA) was pipetted into the glass bottom dish that had been prewarmed at 50 °C. A total of 250 spores in 0.5 μl saline Tween were placed in the middle of a 18 mm cover slip and were left to dry. Cover slips were placed spore‐side down on the non‐solidified CDMGA. When CDMGA had solidified, 2 ml of liquid CD + Met medium (CDMG) was added on top of the culture. Temperature stress was imposed by incubating 2 day old colonies at 4 °C or 45 °C. For pH stress, CDMG of 2 day old cultures was replaced by CDMG with a pH of 2 or 11 adjusted with HCl or NaOH respectively. For hypo‐ and hypertonic conditions, liquid CDMG of 2 day old cultures was replaced with demi water or 1 M MgSO_4_ respectively. Cultures that were C or N‐starved were exposed to C or N‐limiting conditions for 2 days starting at the time of inoculation. To this end, CDMGA was used with 0.2% glucose instead of 1% glucose or CDMGA without methionine respectively. The liquid medium on top of the culture did not contain carbon or nitrogen source. For antibiotics treatment, CDMG and CDMGA were supplemented with either 25 μg ml^−1^ phleomycin or 4 mg ml^−1^ hygromycin. The second compartment (counting from the tip) was ruptured by laser dissection using the laser pressure catapulting function (LPC) of the P.A.L.M. laser dissecting microscope (Carl Zeiss AG; Oberkochen, Germany). To this end, 60%–70% of the power of the pulsed UV‐laser was used. Dissecting the second compartment enabled us to assess the plugging state of the apical septum by monitoring the flow‐out of cytoplasm from the apical to the compartment out of the damaged compartment (Bleichrodt *et al*., 2012). Each experiment was repeated five times for 100 apical septa in total.

### *Recovery of hyphal extension after exposure to stress conditions*

Control and Δ*hexA* strains of *A*. *niger* and *A*. *oryzae* were grown in glass bottom dishes as in the previous section. After 24 h of growth, colonies were either or not exposed to 45 °C or hypertonic conditions for 1,5 h. For C‐starvation, colonies were grown from the moment of inoculation in CDMGA with 0.2% glucose instead of 1% glucose. The liquid medium on top of the culture did not contain carbon source. To recover, liquid medium was replaced by fresh CDMG and left to grow for another 24 h. Hyphal extension within biological triplicates was monitored using P.A.L.M. software and the microscope of the P.A.L.M. laser dissection setup (Carl Zeiss AG; Oberkochen, Germany).

### *Hyphal extension during heat treatment*

Lumox® film (25 μm thickness; Greiner BioOne, Frickenhausen, Germany) was cut into 18 mm diameter circles and 0.5 μl spore solution containing 50,000 spores of *A*. *oryzae* strain RB#140.1 or RB#141.3 was pipetted in the middle of the hydrophobic side of the lumox foil and allowed to dry. Lumox® film was then placed, hydrophobic side down, on a polycarbonate (PC) membrane on MM with 1.5% agarose and supplemented with 200 mM xylose (MMXA). After 2 days of growth at 30 °C, the Lumox® film with the adhering fungal colony was transferred, colony side down, to 500 μl MM supplemented with 25 mM maltose (MMM) in a Cellview™ cell culture dish (Greiner Bio‐One PS, 35/10 MM, 627861) for 4 h at 30 °C. Colonies were then exposed to either 30 °C or 45 °C for 90 min. The cell culture dishes were then placed under the P.A.L.M. laser dissecting microscope (Carl Zeiss AG; Oberkochen, Germany) using a Plan‐Neofluar 40x/0.6 objective and a CCD camera (AxioCam ICc 1, Carl Zeiss AG, Oberkochen, Germany). Fluorescent, growing, leading hyphae were selected, imaged and their growth was recorded for 90 min. Experiments were done in triplicate for each strain and treatment conditions with 30 selected hyphae per replicate.

### *Statistics*

Differences in radial growth between control and treatment conditions were assessed using analysis of variance (ANOVA) with two‐sided Dunnet's post hoc tests or Kruskall–Wallis test followed by pairwise comparisons. Differences in hyphal extension between strains were assessed using ANOVA with Bonferroni post hoc corrections. To asses differences between septal plugging proportions during stress conditions, a Chi‐square test was used. To assess the relation between fluorescence intensity and growth speed, either or not under stress conditions, a test for bimodality was performed a described by and as described by Vinck *et al*. (2005). When populations could be described as bimodal, low fluorescence expressing hyphae were defined as those with a total corrected cellular fluorescence (Burgess *et al*., 2010) ≤$\;{\bar{x}}_{1}$+ its upper 95% confidence interval (CI), intermediate fluorescence expressing hyphae as >$\;{\bar{x}}_{1}$+ its upper 95% CI and <${\bar{x}}_{2}$ – its lower 95% CI and high fluorescence expressing hyphae as ≥${\bar{x}}_{2}$ – its lower 95% CI. Impact of fluorescent hypha location and clustering on hyphal fluorescence intensity was assessed using multinomial logistic regression and Gaussian Mixture Clustering using the R package mclust (Scrucca *et al*., 2016), followed by Spearman's rank correlation respectively. Growth speeds were bootstrapped 1000 times and a three‐way ANOVA was carried out. Differences in fluorescence intensity were analyzed by repeated measures ANOVA or Kruskall–Wallis test. All analyses were carried out in IBM SPSS 24 (IBM Corp. Released 2016. IBM SPSS Statistics for Windows, Version 24.0; IBM Corp., Armonk, NY).

## Conflict of interest

The authors declare no conflict of interest.

## Supporting information


**Supplementary Table 1** Primers used in this study. Underlined sequences indicate *attB* recombination sequences.
