# Supplementary material for: Subpopulations of hyphae secrete proteins or resist heat stress in Aspergillus oryzae colonies
Source: Environ Microbiol. 2019 Nov 24;22(1):447–55. doi: 10.1111/1462-2920.14863 (PMC6972715; doi:10.1111/1462-2920.14863)
Supplement: Supplementary file 1 — Supplementary Table 1 Primers used in this study. Underlined sequences indicate attB recombination sequences. [file EMI-22-447-s001.docx]

**Supplementary Table 1.** Primers used in this study. Underlined sequences indicate *attB* recombination sequences.

| **Primer name** | **Sequence** |
| --- | --- |
| BN088 | ggggacagctttcttgtacaaagtggGCCCACACATCCACCTTCCC |
| BN089 | ggggacaactttgtataataaagttgACAAGGACACCTCCAGCTCTTC |
| BN090 | ggggacaactttgtatagaaaagttgCAGCCCAAGTGGAGAGCGACAAG |
| BN091 | ggggactgcttttttgtacaaacttgCTTGCGAGAAGGAGGGGAATCAA |
| hygroFW | ggggacaagtttgtacaaaaaagcagGCTAGGATTTCGGCACGG |
| hygroREV | ggggaccactttgtacaagaaagctgggtTGTGGAGTGGGCGCTTACAC |
